# Supplementary material for: Identification of Freezing-Responsive microRNAs and Their Targets in Chinese Jujube by Small RNA and Degradome Sequencing
Source: Int J Mol Sci. 2026 Sep 4;27(17):7912. doi: 10.3390/ijms27177912 (PMC13566890; doi:10.3390/ijms27177912)
Supplement: Supplementary file 1 [file ijms-27-07912-s001.zip › Supplementary Material S2/novel_miR_91.pdf]

Provisional ID	:	novel_miR_91
Score total	:	0.7
Score for star read(s)	:	-1.3
Score for read counts	:	0
Score for mfe	:	0.4
Score for randfold	:	1.6
Score for cons. seed	:	
Total read count	:	55
Mature read count	:	55
Loop read count	:	0
Star read count	:	0

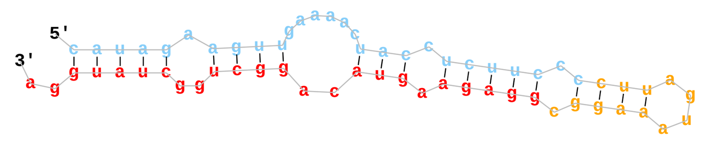

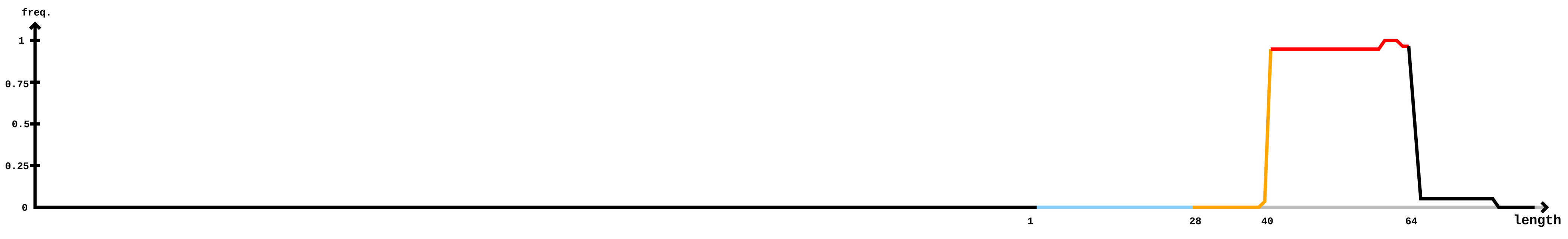

Star Mature

```
5'- ugaaccgcuggcgcagagccagaaagaguaugauaaguaguacaacaaaaauggcaaaaaaaaagaagcaacuuuuuuuuuccccccaaugacugggccaaauuaaauggauaguccaacccgaaccugaaacccgucccuuaccuuggggauucaauugccuacuccauagaaguugaaaacuaccucuuccccuuaguaaaggcggagaaguacaggcuggcuauggaggccaauuggaaagaaaauaaa -3'   exp
    ....(..(((((.....))))).....((((........)))).........((.(((((((((......))))))))).))((((.....)))).........((((...)))).................(((((......)))))((((((((....((((((((.((((......(((.(((((.((((....)))).))))).)))..))))..)))))))).))))))))..)........   reads   mm   sample
    .............................................................................................................................................................................................................Uggagaaguacaggcuggcuaug........................   2   1   LT-1
    ..............................................................................................................................................................................................................ggagaaguacaggcuggcuaugga......................   31   0   LCK-1
    ..............................................................................................................................................................................................................ggagaaguacaggcuggcuCugga......................   1   1   LCK-1
    ..............................................................................................................................................................................................................ggagaaguacaggcuggcuaugga......................   21   0   LCK-2
    .................................................................................................................................................................................................................................auggaggccaauuggaaag........   2   0   JCK-3
    .................................................................................................................................................................................................................................auggaggccaauuggaaag........   1   0   JCK-2
```
